# Supplementary material for: In-Field Detection and Quantification of Septoria Tritici Blotch in Diverse Wheat Germplasm Using Spectral–Temporal Features
Source: Front Plant Sci. 2019 Oct 25;10:1355. doi: 10.3389/fpls.2019.01355 (PMC6824235; doi:10.3389/fpls.2019.01355)
Supplement: Supplementary file 1 [file Table_1.docx]

Supplementary Material

# Supplementary Tables

**Supplementary Table 1** Ranks of spectral-temporal features as determined by recursive feature elimination using random forest (rf) and cubist regression as base learners. Mean feature rank and standard deviation (sd) are reported based on 30 resamples of the data for the top 15 spectral-temporal features. The sensitive SVI (SVI_sen) and insensitive SVI (SVI_insen) constituting the spectral-temporal features and the dynamics parameter used are also reported, where Δ stands for the difference between the parameter values derived from the corresponding SVIs.

| **Spectral-temporal feature** | **Base Learner** | **Mean rank** | **sd** | **SVI_sen** | **SVI_insen** | **Parameter** |
| --- | --- | --- | --- | --- | --- | --- |
| SI_MCARI2-SI_SIPI_r-M_delta | rf | 1.00 | 0.00 | MCARI2 | SIPI | Δ(M) |
| SI_DSWI-SI_780_740-M_delta | rf | 6.17 | 3.81 | DSWI | R780/R740 | Δ(M) |
| SI_VOG1-SI_780_740-M_delta | rf | 6.30 | 4.07 | VOG1 | R780/R740 | Δ(M) |
| SI_MCARI2-SI_780_740-M_delta | rf | 8.50 | 4.23 | MCARI2 | R780/R740 | Δ(M) |
| SI_WI-SI_PRInorm_r-M_delta | rf | 9.03 | 4.13 | WI | PRInorm | Δ(M) |
| SI_MCARI2-SI_PRInorm_r-M_delta | rf | 9.93 | 5.54 | MCARI2 | PRInorm | Δ(M) |
| SI_DSWI-SI_PRInorm_r-M_delta | rf | 11.20 | 5.41 | DSWI | PRInorm | Δ(M) |
| SI_YCAR-SI_FII_r-M_delta | rf | 11.47 | 7.24 | YCAR | FII | Δ(M) |
| SI_VOG1-SI_SIPI_r-M_delta | rf | 12.30 | 5.69 | VOG1 | SIPI | Δ(M) |
| SI_DSWI-SI_GNDVI_HI-M_delta | rf | 12.53 | 3.70 | DSWI | GNDVI | Δ(M) |
| SI_DSWI-SI_SIPI_r-M_delta | rf | 13.27 | 6.60 | DSWI | SIPI | Δ(M) |
| SI_DSWI-SI_DCNI_ASD-M_delta | rf | 13.77 | 6.97 | DSWI | DCNI | Δ(M) |
| SI_MCARI2-SI_SR-b_ratio | rf | 14.53 | 5.47 | MCARI2 | SR | ratio(b) |
| SI_NGRDI-SI_SIPI_r-sen15_delta | rf | 14.97 | 4.56 | NGRDI | SIPI | Δ(sen15) |
| SI_NDWI2130-SI_SIPI_r-sen15_delta | rf | 15.00 | 5.02 | NDWI2130 | SIPI | Δ(sen15) |
| SI_MCARI2-SI_SIPI_r-M_delta | cubist | 1.03 | 0.18 | MCARI2 | SIPI | Δ(M) |
| SI_NGRDI-SI_PRInorm_r-M_delta | cubist | 8.80 | 7.46 | NGRDI | PRInorm | Δ(M) |
| SI_DSWI-SI_780_740-M_delta | cubist | 13.10 | 3.36 | DSWI | R780/R740 | Δ(M) |
| SI_DSWI-SI_FII_r-M_delta | cubist | 13.33 | 4.76 | DSWI | FII | Δ(M) |
| SI_DSWI-SI_GNDVI_HI-M_delta | cubist | 13.50 | 4.16 | DSWI | GNDVI | Δ(M) |
| SI_MCARI2-SI_PRInorm_r-M_delta | cubist | 14.57 | 5.52 | MCARI2 | PRInorm | Δ(M) |
| SI_DSWI-SI_PRInorm_r-M_delta | cubist | 14.67 | 3.52 | DSWI | PRInorm | Δ(M) |
| SI_HI-SI_CHLRE-M_delta | cubist | 14.70 | 3.70 | HI | CHLRE | Δ(M) |
| SI_VI700-SI_CHLRE-M_delta | cubist | 14.77 | 7.67 | VI700 | CHLRE | Δ(M) |
| SI_DSWI-SI_SIPI_r-M_delta | cubist | 15.00 | 3.81 | DSWI | SIPI | Δ(M) |
| SI_MCARI2-SI_780_740-sen15_delta | cubist | 15.13 | 4.99 | MCARI2 | R780/R740 | Δ(sen15) |
| SI_VOG1-SI_780_740-M_delta | cubist | 15.40 | 5.76 | VOG1 | R780/R740 | Δ(M) |
| SI_DSWI-SI_FII_r-sen15_delta | cubist | 15.60 | 2.44 | DSWI | FII | Δ(sen15) |
| SI_NDVI_nb_ASD-SI_GNDVI_HI-M_delta | cubist | 15.63 | 5.54 | NDVI | GNDVI | Δ(M) |
| SI_DSWI-SI_780_740-sen15_delta | cubist | 15.67 | 1.71 | DSWI | R780/R740 | Δ(sen15) |

# Supplementary Figures


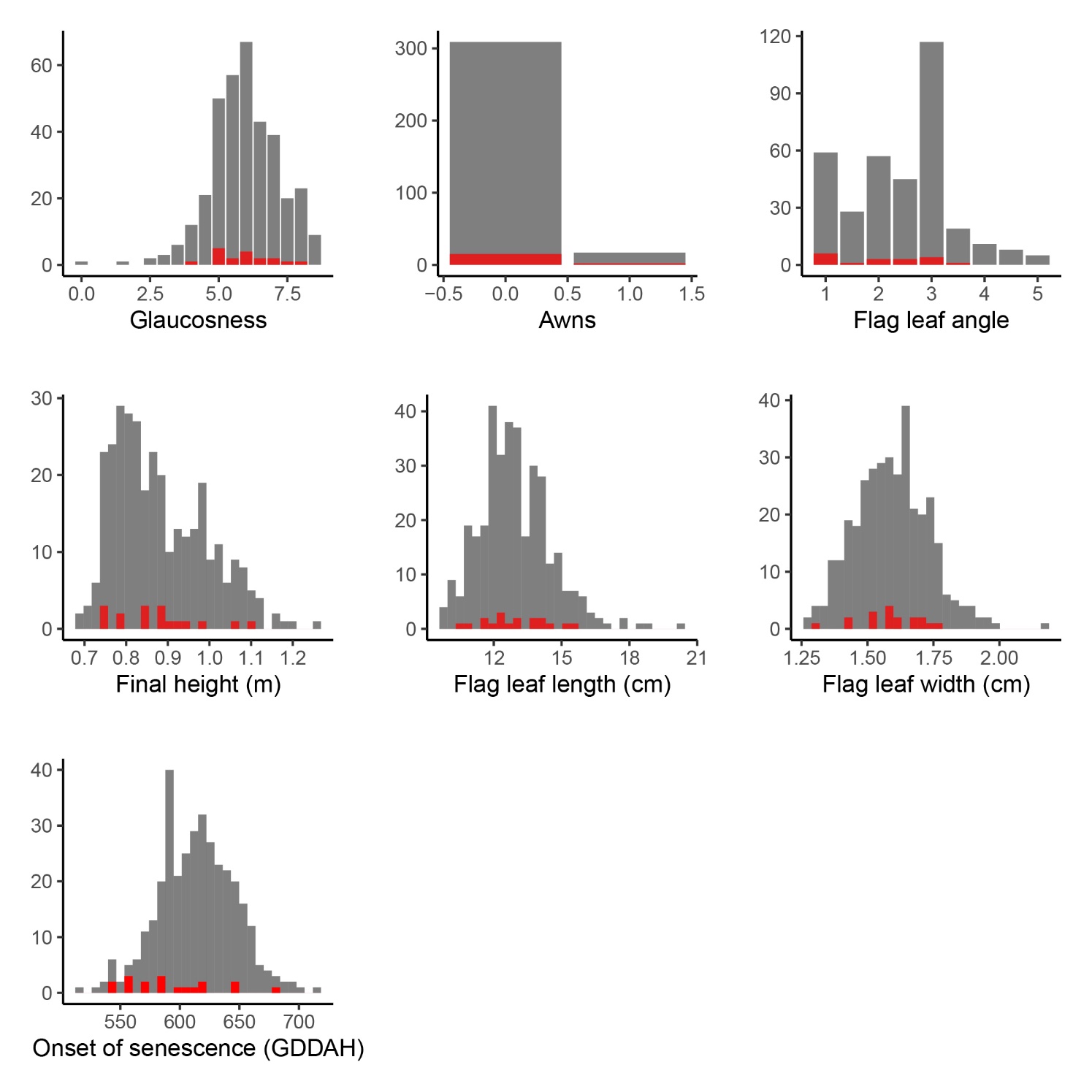


# Supplementary Figure 1 Distribution of important morphological, phenological and canopy structural traits in the GABI wheat panel and in the subset of genotypes selected for the present study. Flag leaf glaucousness, presence or absence of awns, flag leaf angle, flag leaf length and flag leaf width were assessed in 2018, following guidelines provided by Pask et al. (2012). For Final height and onset of senescence, distributions of best linear unbiased estimators from experiments conducted in three consecutive years at the same location are shown. For details on methods to determine these traits, we refer to Anderegg et al. (2019), Kronenberg et al. (2017) and Pask et al. (2012).
